# Salvation—Health

**Published:** 1875-04

**Authors:** Thos. K. Beecher


### SALVATION--HEALTH.

THOS. K. BEECHER.

I have before me the New Testament translated by WILLIAM TYNDALL, and published (so the title page declares), in 1526. I find that the word translated—"*salvation*"—in our familiar Testament, TYNDALL oftener, though not invariably, renders "*health.*" As, for instance, [Titus 2:11] "The grace of God which bringeth *health* hath appeared to all men."

Salvation and health are indeed nearer of kin than most men have supposed. Religion and hygeine, to the truly comprehensive mind, cover about the same ground. It was an unfortunate day when the cure of the soul was set off to pastors and priests, and the cure of the body to physicians and surgeons. What God hath joined together let no man put asunder.

Opening the Old Testament, and studying attentively the law as given by Moses, we find it almost impossible to draw a line between the precepts that are strictly moral and religious, and other precepts that are clearly hygienic. It will be found that many of the minute directions given by Moses as to personal habits and the policing of the camp, have a foundation which modern sanitary science makes haste to recognize. And before the days of Moses, in Egypt, the priests and the physicians were one and the same set of men.

Jesus, too, was chiefly known to the men of his generation as a healer of diseases. He accepted this reputation. When John enquired whether he were indeed the Son of God, he replied: See! the blind receive sight; the lame walk; the lepers are cleansed; the deaf hear; the dead are raised, and the poor have the gospel preached to them. This was a consistent part of his work. He who undertakes to save from sin must necessarily save from the wages of sin, which are sickness and death. Healing a paralytic he said: "Thy sins are forgiven thee," and with forgiveness came health.

After the days of Jesus Christ upon earth, the cure of diseases was a frequent part of the ministry of the apostles and early missionaries. Luke, chiefly known to us as a biographer, was chiefly known in his day as a physician. In times of persecution, thousands of wandering christians were sheltered from injury by their holy calling as healers of disease. The saints, a glorious company, whose works ornament the history of the Catholic church, almost without exception, won gratitude and immortal fame by their works of usefulness and healing. This very day, too, the most successful foreign missionaries that have been sent out by any board, are they who, in addition to the good news of eternal life, have gained some skill as physicians and healers of all manner of diseases.

The entire drift of scripture, tradition and experience, is in the direction of salvation or health to the *whole man*. It is only when we come to comparatively modern days that we find body divorced from soul, and a profession growing up that endeavors to treat the body scientifically; and another profession that endeavors to treat the soul theologically; the one vainly endeavoring health for men *until* they die, and the other guaranteeing health to them *after* they die.

We doubt that either profession can fulfil the guarantee, except the members in each take into account the domain claimed by the other. As a physician he is a blind bungler who does not recognize again and again ailments that can be relieved only by a change of affections, or, as we preachers say, "a new heart." Equally blind and bungling is that pastor who fails to note again and again intense passions of one kind and another that have their real fountain in the flesh and not in the spirit.

I note that there are four professions that have as their "raw material" to work upon human nature—teachers, doctors, lawyers and pastors. And of these four only one seems to me to be making full proof of its ministry. This one (the teachers), takes human nature at the teachable age, and guides it so as to avoid, if possible, damage, and attain growth. The other three are called upon chiefly in the hour of disaster, to remedy evils that are always more or less remediless. The lawyer comes in when the peace is broken, to help the parties fight out their quarrel under the law. The doctor comes in when health is gone, to help his patient back to the lost path. The preacher comes in to teach repentence and return to God.

It would seem as if there were a department of possible usefulness neglected by doctors, preachers and lawyers, namely, the department of teaching men; doctors teaching how to keep well, preachers teaching how to retain a clean conscience, lawyers how to be at peace with all men. An ounce of prevention is worth a pound of cure. That community is to be envied and imitated in which may be found doctors, lawyers and pastors working together as one man for the salvation that is the health of body, spirit and society.

NEW PAPER.—How do you like our tinted paper, also our bran-new type?

